# Ningmitai capsule in patients with chronic prostatitis/chronic pelvic pain syndrome: a multicenter, prospective, randomized, parallel, positive-controlled study

**DOI:** 10.3389/fphar.2025.1667819

**Published:** 2025-12-03

**Authors:** Jingjing Gao, Yao Zhang, Junhua Du, Qiang Liu, Yi Cheng, Wentao Yang, Daoyuan Wang, Wei Wang, Liang Zheng, Dong Wang, Lixin Wu, Xiaolei Jiang, Qunli Men, Chaozhao Liang, Xiansheng Zhang

**Affiliations:** 1 Department of Urology, First Affiliated Hospital of Anhui Medical University, Hefei, Anhui, China; 2 Department of Urology, 9 O 3 Hospital, Jiangyou, Sichuan, China; 3 Department of Andrology, First Affiliated Hospital of Anhui University of Traditional Chinese Medicine, Hefei, Anhui, China; 4 Department of Andrology, Ruikang Hospital Affiliated to Guangxi University of Chinese Medicine, Nanning, Guangxi, China; 5 Department of Urology, 989 Hospital of Joint Service Support Force of Chinese People’s Liberation Army, Luoyang, Henan, China; 6 Department of Urology, Hefei Second People’s Hospital, Hefei, Anhui, China; 7 Department of Urology, Second Affiliated Hospital of Xi’an Medical College, Xi’an, Shaanxi, China; 8 Department of Urology, Huantai Branch, Qilu Hospital, Zibo, Shandong, China; 9 Department of Urology, Hefei Third People’s Hospital, Hefei, Anhui, China; 10 Department of Urology, Mianyang 404 Hospital, Mianyang, Sichuan, China; 11 Department of Urology, Baoji Central Hospital, Baoji, Shaanxi, China

**Keywords:** chronic prostatitis/chronic pelvic pain syndrome, ningmitai capsule, NIH-CPSI, QoL, multimodal therapy

## Abstract

**Background:**

CP/CPPS is characterized by pain, the primary symptom, which significantly affects QoL and disrupts lower urinary tract function. Conventional monotherapies are often ineffective, necessitating multimodal treatments targeting key symptoms.

**Methods:**

This multicenter, prospective, randomized, parallel, positive-controlled trial enrolled 323 men aged 18–60 years with CP/CPPS across 14 centers in China. Participants were randomly assigned to three groups: tamsulosin 0.2 mg daily, Ningmitai capsule (NMT) 1.52 g thrice daily, or a combination of both, for 8 weeks. The primary endpoint was the change in the total NIH-CPSI score from baseline to week 8. Secondary endpoints included changes in pain, urinary, QoL subdomains, the percentage of patients achieving a ≥25% reduction in NIH-CPSI total score, and pain scores. Safety was monitored through adverse events and liver function tests.

**Results:**

Of the 323 patients, 108 received tamsulosin, 109 NMT, and 106 combination therapy. After 8 weeks, both NMT and combination therapy demonstrated significantly greater reductions in total NIH-CPSI scores compared to tamsulosin (−11.44 vs. −8.58, *P* < 0.001; −11.94 vs. −8.58, *P* < 0.001). Combination therapy also significantly reduced pain (*P* = 0.001) and improved QoL (*P* < 0.001) compared to tamsulosin. The NMT group showed greater improvements in pain scores at both 4 and 8 weeks compared to tamsulosin (*P* < 0.05). At week 8, the percentage of patients achieving a ≥25% reduction in NIH-CPSI total score was significantly higher in the NMT (78.64% vs. 55.91%, *P* < 0.001) and combination groups (82.83% vs. 55.91%, *P* < 0.001). Subgroup analyses indicated that NMT was most effective in patients with mild to moderate CP/CPPS, younger age (18–34 years), and disease duration of <12 months. No serious adverse events occurred, and NMT was well-tolerated across all groups.

**Conclusion:**

NMT demonstrated superior efficacy over tamsulosin in reducing pain and improving QoL in CP/CPPS patients. Combination therapy provided enhanced symptom relief, particularly in micturition and QoL domains, supporting a multimodal approach for more effective CP/CPPS management. These findings validate NMT as a promising treatment, either alone or in combination, for CP/CPPS. Further long-term studies are needed to optimize its use.

**Clinical Trial Registration:**

ClinicalTrials.gov, identifier NCT05890235.

## 1 Introduction

Prostatitis is a prevalent and costly urological disorder, with the prevalence of prostatitis-like symptoms ranging from 2.2% to 16% in different studies (Hua et al., 2025; Sugimoto et al., 2022; Liang et al., 2009). Among its subtypes, chronic prostatitis/chronic pelvic pain syndrome (CP/CPPS), the most frequent subtype (≥90%) encountered by urologists, is the occurrence of chronic pain when there is no proven infection or other obvious local pathology that may account for the pain (Pena et al., 2021; Meng et al., 2022; Snipes et al., 2025). Beyond pain, CP/CPPS often leads to urinary and sexual dysfunction, and can significantly impact patients’ negative cognitive, behavioural, or emotional wellbeing (Zhang et al., 2020; Yebes et al., 2023).

Pain is the primary symptom of CP/CPPS, and its complex and multifactorial etiology makes monotherapy insufficient (Polackwich and Shoskes, 2016; Zaidi et al., 2018). Most patients require multimodal treatment aimed at the main symptoms, and taking comorbidity into account, which are consistent with the traditional Chinese medicine (TCM) theory for treatment of CP/CPPS based on syndrome differentiation and overall concept (Fall et al., 2010; Sun et al., 2021; Xue et al., 2019).

TCMs, typically derived from a combination of herbal ingredients, offer the potential for multi-target and multi-mechanism therapeutic effects, addressing various symptoms simultaneously (M et al., 2023). Ningmitai capsule (NMT), a TCM derived from seven medical herbs, is approved for treating chronic prostatitis and urinary tract infections, among other urogenital diseases. Clinical trials have shown NMT to offer significant therapeutic benefits. A systematic review and meta-analysis of data from 30 uncontrolled CP/CPPS studies including 6,185 patients indicated that the use of NMT provided a statistically significant clinical benefit (Jin et al., 2018). This existing evidence supports the need for further rigorous evaluation of NMT, thus motivating the current multicenter, randomized, positive-controlled trial to definitively assess its efficacy and safety in patients with abacterial CP/CPPS. Further mechanistic studies have revealed that NMT, particularly through components such as quercetin, exerts anti-inflammatory and pain-relieving effects in CP/CPPS animal models by modulating systemic and local immune responses, including the inhibition of the MAPK, NF-κB, and STAT3 pathways and reduction of CCL2 secretion (Liu et al., 2022).

Base on this recognized clinical application and existing evidence, the present multicenter, randomized, positive-controlled trial aims to evaluate the efficacy and safety of 8 weeks of NMT therapy compared with tamsulosin in patients with mild to severe abacterial CP/CPPS, irrespective of prior α-blocker treatment, providing further high-quality evidence for its role in managing this challenging condition.

## 2 Methods

### 2.1 Participants

Study participants were recruited from 14 centers across China, the first subject first visit was 16 November 2018 and the last subject last visit was 10 July 2022. Eligible participants were men 18–60 years of age with CP/CPPS who had a National Institutes of Health Chronic Prostatitis Symptom Index (NIH-CPSI) pain score of at least 4 at screening, had experienced pain or discomfort in the pelvic region for at least 3 months during the previous 6 months.

Exclusion criteria included a documented history of genitourinary cancer, prostate or bladder surgery, or a urinary tract infection (defined as a midstream urine culture ≥100,000 colonies/mL) within the past 3 months. Patients with liver or renal insufficiency were also excluded. To prevent confounding, all participants were required to discontinue antimicrobial agents, α-adrenergic receptor blockers, analgesic medications, phytotherapy, or other Traditional Chinese Medicines for a minimum of 2 weeks prior to enrollment. No confounding medications or concomitant treatments were permitted throughout the study duration. All eligible participants provided written informed consent.

### 2.2 Study design

This 8-week, multicenter, prospective, randomized, parallel, positive-controlled study (ClinicalTrials.gov identifier NCT05890235) was conducted according to the Declaration of Helsinki and Good Clinical Practice (GCP) guidelines. The study protocol was approved by the ethical committee of Clinical Medical Research of Anhui Medical University’s First Affiliated Hospital (Ethical Approval No.: PJ2018-13-13).

At the screening phase, a comprehensive medical history, physical examination, digital rectal examination, and a lower urinary tract two-glass test was performed (Seiler et al., 2003). Eligible patients were randomized 1:1:1 to one of three treatment arms: tamsulosin alone (0.2 mg once daily), NMT alone (1.52 g thrice daily; Guiyang Xintian Pharmaceutical Co., Ltd., China; Lot: 181201), or a combination of both drugs for 8 weeks. NMT is a commercially available Traditional Chinese Medicine (TCM) manufactured by Guiyang Xintian Pharmaceutical Co., Ltd. The capsule is formulated to clear heat, relieve stranguria, and dispel moisture and toxins. Approved by the National Medical Products Administration (NMPA) in 2002 (Approval Number: Z20025442), it is indicated for the treatment of chronic prostatitis, urinary tract infections, and other urogenital system diseases, with its clinical efficacy being widely recognized. The capsule is composed of seven botanical constituents, with their specific components and proportions detailed in Table 1. The formulation adheres to the TCM compatibility principle of “sovereign, minister, assistant, and guide”: *Polygonum capitatum Buch.-Ham. ex D. Don Prodr* (Touhualiao, 30%), *Imperata cylindrica Beauv. var. major (Nees) C. E. Hubb.* (Baimaogen, 17%), *Cocculus orbiculatus C. K. Schneid.* (Dafengteng, 15%), *Berberis soulieana Schneid. and Berberis wilsonae Hemsl.* (Sankezhen, 11%), *Agrimonia pilosa Ledeb.* (Xianhecao, 11%), *Hibiscus mutabilis L.* (Mufurongye, 1%), and *Forsythia suspensa (Thunb.) Vahl* (Lianqiao, 15%).

**TABLE 1:** Botanical composition and source information of Ningmitai Capsule.

Full botanical drug species name	Chinese botanical drugs name	Proportion
Polygonum capitatum Buch.-Ham. ex D. Don Prodr	Touhualiao	30%
Imperata cylindrica Beauv. var. major (Nees) C. E. Hubb.	Baimaogen	17%
Cocculus orbiculatus C. K. Schneid.	Dafengteng	15%
Berberis soulieana Schneid. and Berberis wilsonae Hemsl.	Sankezhen	11%
Agrimonia pilosa Ledeb.	Xianhecao	11%
Hibiscus mutabilis L.	Mufurongye	1%
Forsythia suspensa (Thunb.) Vahl.	Lianqiao	15%

As for the preparation of NMT, *A. pilosa*, *F. suspensa*, and *I. cylindrica* (77 g each) were pulverized into fine powder, passed through a standard sieve, and thoroughly mixed. The remaining Agrimonia pilosa, Forsythia suspensa, Imperata cylindrica, and the other four crude drugs were decocted three times with water—first for 2 h, and subsequently twice for 1.5 h each. The combined decoctions were filtered and concentrated under reduced pressure to a relative density of 1.15–1.21 at 60 °C–70 °C to yield a soft extract. The extract was blended with the pre-prepared fine powder, dried, pulverized, and encapsulated to obtain the final product. Pharmaceutical quality control was ensured following the ConPhyMP (Consortium on Phytomedicine and Phytotherapy) best-practice framework (Heinrich et al., 2022). Each capsule complies with the Chinese national drug standard WS-10348-(ZD-0348)2002-2012Z, containing a minimum of 1 mg gallic acid as specified.

Tamsulosin, an α-adrenergic receptor blocker, was chosen as a comparator given its widespread use in CP/CPPS treatment and its demonstrated moderate but significant beneficial effects (Chen et al., 2011; Kim et al., 2011; Lai et al., 2025a; Lai et al., 2025b).

Each patient was randomly assigned by Interactive Response Technology (IRT) system that enables patient randomization, real-time drug allocation, and dynamic drug supply forecasting. A permuted-block randomization procedure with randomly assigned block sizes of 6 was used, stratified by clinical site. Although participants could potentially distinguish between treatment groups due to the differing appearance and taste of NMT capsules and tamsulosin tablets, investigators, care providers, and those assessing clinical outcomes were blinded to treatment assignments throughout the trial. Furthermore, statisticians remained blinded to the treatment assignment during data analysis.

The study involved four scheduled research-clinic visits: visit 1 encompassed screening procedures, visit 2 involved randomization, drug dispensation, and collection of baseline data. Subsequent visits at week 4 (visit 3) and week 8 (visit 4) focused on evaluating efficacy and safety outcomes.

### 2.3 Outcomes

The efficacy assessments were made with self-administered patient surveys, and patients completed the National Institutes of Health Chronic Prostatitis Symptom Index (NIH-CPSI) and subscales at baseline, weeks 4 and 8 of the study. The NIH-CPSI consists of three domains (nine questions on a scale of 0–43): pain (score 0–21), voiding disturbances (score 0–10), and quality of life (score 0–12) (Litwin et al., 1999). The primary end point was change from baseline to week 8 in the NIH-CPSI total score. Secondary end points included percentages of responders, change from baseline in the NIH-CPSI pain, urinary, quality of life (QoL) subscores, and safety. The responders were predefined as patients who had experienced a 25% decrease in the total NIH-CPSI score (total score responders) and/or NIH-CPSI pain subscore (pain subscore responders) from the baseline values (Franco et al., 2019; Nickel et al., 2003).

Safety was monitored during the whole course of the study through vital signs assessments, clinical laboratory tests and adverse events reporting. The investigator evaluated the adverse events as to their severity and relationship to the investigational drug according to the International Council for Harmonisation of Technical Requirements for Pharmaceuticals for Human Use (ICH) harmonised guideline E3: Structure and Content of Clinical Study Reports. All adverse events were categorized and reported in accordance with the codes in the Medical Dictionary for Regulatory Activities (MedDRA), version 27.0, whether or not they were considered to be related to the investigational drug.

### 2.4 Detection of active components in NMT

#### 2.4.1 Sample preparation

The desiccated plant samples were ground into a fine powder. A 50 mg portion of the powder was accurately weighed, mixed with beads, and extracted with 500 µL of an extraction solution composed of methanol:acetonitrile:water (2:2:1, v/v/v) containing isotope-labeled internal standards. The solution was vortexed for 30 s, homogenized (35 Hz for 240 s), and then sonicated for 5 min in a 4 °C water bath. This homogenization-sonication cycle was repeated a total of three times. The mixture was then incubated for 30 min at −40 °C to facilitate protein precipitation. Subsequently, the samples were centrifuged at 12,000 rpm (13,800 × g) for 15 min at 4 °C. The supernatant was transferred to a fresh vial, incubated for 10 min, and centrifuged again under the same conditions. The final supernatant was collected and passed through a 0.22 µm microfilter before being transferred to a glass vial for analysis. A quality control (QC) sample was prepared by pooling equal aliquots of the supernatant from each experimental sample to monitor system stability and repeatability.

#### 2.4.2 LC-MS/MS analysis

Metabolite analysis was performed using an ultra-high performance liquid chromatography (UHPLC) system (Vanquish, Thermo Fisher Scientific) coupled to an Orbitrap Exploris 120 mass spectrometer (Thermo Fisher Scientific). Chromatographic separation was achieved on a Phenomenex Kinetex C18 column (2.1 mm × 100 mm, 2.6 µm). The mobile phase consisted of solvent A (0.01% acetic acid in water) and solvent B (isopropanol:acetonitrile, 1:1, v/v). The autosampler temperature was maintained at 4 °C, and the injection volume was 2 µL.

The Orbitrap Exploris 120 mass spectrometer was operated in data-dependent acquisition (DDA) mode. The electrospray ionization (ESI) source parameters were as follows: sheath gas flow rate, 50 Arb; auxiliary gas flow rate, 15 Arb; capillary temperature, 320 °C. Full MS scans were acquired with a resolution of 60,000, and MS/MS scans were acquired with a resolution of 15,000. Collision energy was set to SNCE 20/30/40, and the spray voltage was 3.8 kV in positive mode and −3.4 kV in negative mode.

#### 2.4.3 Data processing and annotation

The raw data were converted to the mzXML format using ProteoWizard and processed with an in-house program. The R package and the BiotreeDB (V3.0) were applied in metabolite identification.

### 2.5 Statistical analysis

All statistical analyses were conducted using SPSS version 23.0. Comparison between groups and within groups of the NIH-CPSI total and domain scores changes were evaluated by Analysis of Covariance (ANCOVA), with baseline NIH-CPSI score as a covariate. Last Observation Carried Forward (LOCF) was used to handle missing values in NIH-CPSI scores. Response would be regarded as 0 when there were missing values in NIH-CPSI scores before LOCF. The response rate between groups was analyzed using χ^2^ test. Furthermore, explorative analysis was conducted to see the treatment impact on NIH-CPSI response rate by using logistic regression. Additionally, demographics, baseline conditions, and safety data were analyzed. Method for two-group comparison were employed: ANCOVA or nonparametric test, paired-t test, χ^2^ test, and Fisher’s exact probability method.

Multiplicity management: firstly, a hypothesis was tested to see whether the combination group was superior than tamsulosin group. If null hypothesis has been rejected, the next hypothesis testing would be performed to see whether NMT group was superior than tamsulosin group. If null hypothesis has been rejected too, clinical superiority can be claimed. In the test of combination group versus tamsulosin, a total of sample 106 participants would be required under treatment difference of −3.5 points with 5.5 SD, α = 0.05 and β = 0.9 (Jin et al., 2018). In the test of NMT group versus tamsulosin, a total of sample 144 participants would be required under treatment difference of −3 points with 5.5 SD, α = 0.05 and β = 0.9. Combining the hypothetical sample size estimates for the two tests, a minimum of 270 subjects including 20% dropouts would be included, with a 1:1:1 ratio in each group.

## 3 Results

### 3.1 Chemical profiling of NMT

The pharmacological activity of NMT is attributed to its complex chemical composition. As a TCM formulation, the sample contains a large number of chemical components, primarily derived from the secondary metabolites of the herbal ingredient (Supplementary Figure S1).

### 3.2 Study participants

**FIGURE 1:**
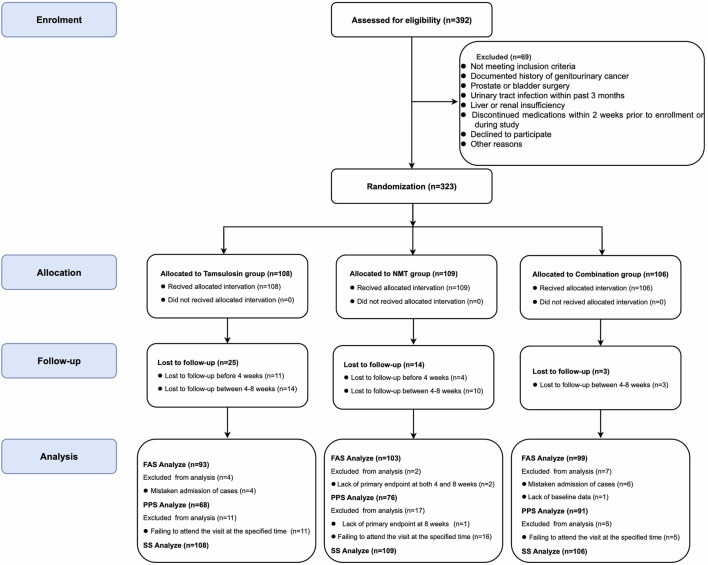
CONSORT (consolidated standards of reporting trials) flowchart for study participants.

A total of 392 individuals with CP/CPPS were screened for this study. Of these, 69 were excluded for various reasons, including failure to meet the inclusion criteria or refusal to participate. Ultimately, 323 patients were randomly assigned (1:1:1) to tamsulosin (n = 108), NMT (n = 109), or combination (n = 106) group. The research flow chart is shown in Figure 1.

During the trail, 10 subjects were mistakenly included, 15 subjects dropped out before 4 weeks of treatment, 27 subjects dropped out between 4 and 8 weeks, 3 subjects lost NIH-CPSI total score data at baseline, 1 subject lost NIH-CPSI total score data at 8 weeks, and 32 subjects exceeded the visiting window. As a result, 295 participants were included in the full analysis set (FAS) and 235 in the per-protocol set (PPS).

Table 2 shows the baseline characteristics of each group. The average duration of CP/CPPS symptoms among them was more than 16 months. At baseline, there was no significant difference in the three groups’ average NIH-CPSI total score, urinary score, (*P* > 0.05).

**TABLE 2:** Patient demographics and baseline characteristics^a^.

Characteristics	Tamsulosin group	NMT group	Combination group	P-value
No. of patients	93	103	99	
Age (yr)	36.09 ± 10.30	36.37 ± 10.03	32.83 ± 8.17	0.013
Age, no. (%)				0.018
18–34	42/93 (45.16)	45/103 (43.69)	63/99 (63.64)	
35–44	29/93 (31.18)	33/103 (32.04)	26/99 (26.26)	
≥45	22/93 (23.66)	25/103 (24.27)	10/99 (10.10)	
Region, no. (%)				<0.001
Southern China	87/93 (93.55)	89/103 (86.41)	71/99 (71.72)	
Northern China	6/93 (6.45)	14/103 (13.59)	28/99 (28.28)	
Classification of prostatitis, no. (%)†				<0.001
IIIA	30/93 (32.26)	50/103 (48.54)	32/99 (32.32)	
IIIB	43/93 (46.24)	34/103 (33.01)	56/99 (56.57)	
Unknown	20/93 (21.51)	19/103 (18.45)	11/99 (11.11)	
Duration of disease (mth)	18.06 ± 21.77	19.17 ± 35.44	16.39 ± 16.73	0.270
NIH-CPSI				
Total score (0–43)	23.20 ± 6.40	24.10 ± 7.02	24.70 ± 5.02	0.251
Pain score (0–21)	9.68 ± 3.16	10.31 ± 3.36	10.85 ± 3.08	0.045
Urinary score (0–10)	5.48 ± 2.35	5.40 ± 2.49	4.66 ± 2.44	0.053
Quality-of-Life (QoL) score (0–12)	8.04 ± 2.34	8.39 ± 2.50	9.13 ± 1.84	0.003

^a^ Plus-minus values are means ± SD.

NMT: ningmitai capsule, Combination: Ningmitai capsule+tamsulosin.

†IIIA, prostatitis refers to the white blood cell count >10/HP, in expressed prostatic screen, and IIIB, means white blood cell count ≤10/HP., In the FAS analysis set, 245 subjects received the expressed prostatic section examination.

### 3.3 Study end points

#### 3.3.1 Primary efficacy outcomes

**FIGURE 2:**
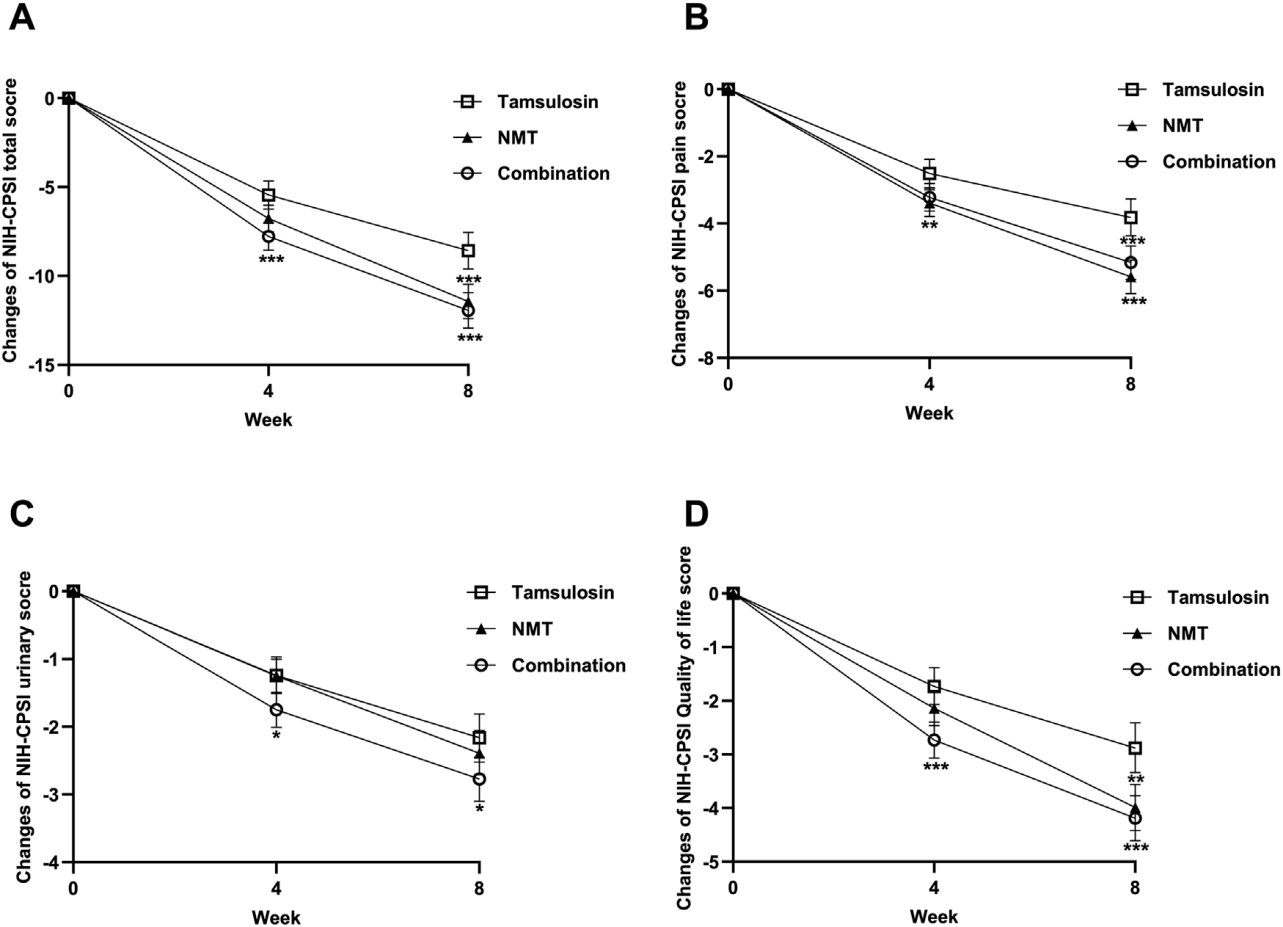
Changes in NIH-CPSI Score from Baseline to 4 and 8 Weeks. **(A)** Changes in NIH-CPSI total score; **(B)** Changes in NIH-CPSI pain score; **(C)** Changes in NIH-CPSI urinary score; **(D)** Changes in NIH-CPSI Quality of Life score. The top and bottom of the vertical line represent the 95% confidence interval at 4 and 8 weeks. NMT = Ningmitai capsule; Combination = Ningmitai capsule + Tamsulosin. Compared with the tamsulosin group, *P* < 0.05, *P* < 0.01, *P* < 0.001.

The primary efficacy endpoint, defined as the change in total NIH-CPSI score from baseline to 8 weeks, is presented in Table 3 and Figure 2A. Patients receiving combination therapy demonstrated a significant decrease in total NIH-CPSI score compared to those treated with tamsulosin alone (−11.94 vs. −8.58, P < 0.001), confirming our superiority hypothesis. We then tested the non-inferior hypothesis that the total NIH-CPSI score of NMT group was not inferior to that of tamsulosin group. Decreases in total NIH-CPSI scores was also significant higher with NMT than tamsulosin (−11.44 vs. −8.58, *P* < 0.001). The results from the PPS were generally consistent with those from the FAS.

**TABLE 3:** Total score changes at 8 weeks in NIH-CPSI for primary outcome.

NIH-CPSI score		Estimate in ANCOVA model (95% *CI*)^a^			Combination VS. Tamsulosin		NMT VS. Tamsulosin		Combination VS. NMT	
		Tamsulosin	NMT	Combination	Difference (95% *CI*)	P^b^	Difference (95% *CI*)	P^b^	Difference (95% *CI*)	P^b^
Total score										
Week 8	FAS	−8.58 (−9.61, −7.56)	−11.44 (−12.41, −10.47)	−11.94 (−12.93, −10.94)	−3.36 (−5.11, −1.60)	<0.001	−2.86 (−4.59, −1.13)	<0.001	−0.50 (−2.20, 1.20)	1.000
	PPS	−8.58 (−9.76, −7.41)	−11.48 (−12.59, −10.37)	−11.80 (−12.81, −10.78)	−3.21 (−5.12, −1.31)	<0.001	−2.90 (−4.88, −0.92)	0.001	−0.31 (−2.16, 1.53)	1.000

^a^ Analysis of Covariance (ANCOVA) model was used, with baseline score as covariate.

^b^ Bonferroni method was used to adjust the test level between groups.

NMT: ningmitai capsule, Combination: Ningmitai capsule+tamsulosin.

#### 3.3.2 Secondary efficacy outcomes

Analyses of secondary efficacy endpoints further supported the findings of the primary efficacy analysis (Table 4).

**TABLE 4:** Total and domain score changes at 4 and 8 weeks in NIH-CPSI for secondary outcome.

Outcome	Tamsulosin	NMT	Combination	Combination VS. Tamsulosin		NMT VS. Tamsulosin		Combination VS. NMT	
NNIH-CPSI	Estimate mean change (95% *CI*)^a^			Estimate mean difference (95%*CI*)^a^ and P-value^b^					
Total score6									
Week 4	−5.45 (−6.24, −4.66)	−6.77 (−7.53, −6.02)	−7.78 (−8.55, −7.01)	−2.33 (−3.69, −0.98)	<0.001	−1.32 (−2.66, 0.01)	0.053	−1.01 (−2.32, 0.31)	0.197
Week 8	−8.7 (−13.22, 2.82)	−11.92 (−24.25, 0.41)	−12.38 (−22.77, 1.99)	−3.40 (−4.84, −1.97)	<0.001	−2.88 (−4.35, −1.41)	<0.001	−0.18 (−1.54, 1.19)	0.799
Pain score									
Week 4	−2.51 (−2.94, −2.09)	−3.39 (−3.79, −2.99)	−3.22 (−3.63, −2.81)	−0.71 (−1.43, 0.01)	0.056	−0.88 (−1.59, −0.17)	0.009	0.17 (−0.53,0.87)	1.000
Week 8	−3.82 (−4.36, −3.27)	−5.59 (−6.09, −5.09)	−5.16 (−5.66, −4.67)	−1.35 (−2.25, −0.45)	0.001	−1.77 (−2.68, −0.87)	<0.001	0.43 (−0.44,1.29)	0.706
Urinary score									
Week 4	−1.24 (−1.50, −0.97)	−1.25 (−1.51, −1.00)	−1.75 (−2.01, −1.49)	−0.51 (−0.98, −0.05)	0.023	−0.02 (−0.47, 0.44)	1.000	−0.50 (−0.95, −0.05)	0.024
Week 8	−2.16 (−2.52, −1.81)	−2.39 (−2.71, −2.06)	−2.77 (−3.10, −2.45)	−0.61 (−1.20, −0.02)	0.040	−0.22 (−0.81, 0.37)	1.000	−0.39 (−0.95,0.17)	0.292
QoL score									
Week 4	−1.73 (−2.07, −1.38)	−2.14 (−2.46, −1.81)	−2.73 (−3.07, −2.40)	−1.01 (−1.60, −0.41)	<0.001	−0.41 (−0.99, 0.17)	0.264	−0.60 (−1.17, −0.03)	0.037
Week 8	−2.88 (−3.34, −2.41)	−3.99 (−4.42, −3.56)	−4.19 (−4.61, −3.77)	−1.32 (−2.09, −0.54)	<0.001	−1.11 (−1.88, −0.34)	0.002	−0.20 (−0.94,0.54)	1.000

^a^ Analysis of Covariance (ANCOVA) model was used, with baseline score as covariate.

^b^ Bonferroni method was used to adjust the test level between groups.

NMT: ningmitai capsule, Combination: Ningmitai capsule+tamsulosin.

At 4 weeks, the tamsulosin group had a 5.45 points mean decrease in the total score for the NIH-CPSI, as compared with a 7.78-points mean decrease in the combination group (*P <* 0.001) and 6.77 points mean decrease in the NMT group (*P* = 0.053), respectively (Figure 2A).

Further analysis of the NIH-CPSI pain domain revealed statistically significant treatment effects for the combination group compared to the tamsulosin group at 8 weeks (−5.16 vs. −3.82, *P* = 0.001, Figure 2B). Notably, the NMT group also showed a significantly higher improvement in the pain symptom score compared to the tamsulosin group at both 4 weeks (−3.39 vs. −2.51, *P* = 0.009) and 8 weeks (−5.59 vs. −3.82, *P* < 0.001).

Improvements in the micturition domain of the NIH-CPSI were observed across all treatment groups. There was a significant improvement between the combination group and the tamsulosin group in the change from baseline to week 4 (−1.75 vs. −1.24, *P* = 0.023) and week 8 (−2.77 vs. −2.16, *P* = 0.040, Figure 2C). All groups exhibited progressive improvement in QoL scores. Combination therapy provided significantly greater improvement in QoL scores compared to tamsulosin monotherapy (−2.73 vs. −1.73 at 4 weeks, *P* < 0.001; −4.19 vs. −2.88 at 8 weeks, *P* < 0.001, Figure 2D).

#### 3.3.3 Responder analyses

Responder analyses, defined by a ≥25% reduction in total NIH-CPSI score from baseline, demonstrated significant differences among treatment groups (Table 5). At 4 and 8 weeks, the proportion of responders was 44.09% and 55.91% for the tamsulosin group, respectively. For the NMT group, these proportions were 54.37% (4 weeks, P = 0.151) and 78.64% (8 weeks, *P* < 0.001). The combination group showed 68.69% responders at 4 weeks (*P* < 0.001 vs. tamsulosin) and 82.83% at 8 weeks (*P* < 0.001).

**TABLE 5:** Percentage of patients with 25% decrease in total NIH-CPSI score and pain score at 4 and 8 weeks.

Response rate	Treatment			*P* value			
	Tamsulosin	NMT	Combination	P	Combination VS. Tamsulosin^a^	NMT VS. Tamsulosin^a^	Combination VS. NMT^a^
NIH-CPSI pain score decreased by 25%, N (%)							
Week 4	40/93 (43.01)	67/103 (65.05)	69/99 (69.70)	<0.001	<0.001	0.002	0.481
Week 8	48/93 (51.61)	85/103 (82.52)	84/99 (84.85)	<0.001	<0.001	<0.001	0.656
NIH-CPSI total score decreased by 25%, N (%)							
Week 4	41/93 (44.09)	56/103 (54.37)	68/99 (68.69)	0.003	<0.001	0.151	0.037
Week 8	52/93 (55.91)	81/103 (78.64)	82/99 (82.83)	<0.001	<0.001	<0.001	0.451

^a^ Bonferroni correct at α = 0.0167 was used.

NMT, ningmitai capsule, Combination = Ningmitai capsule+tamsulosin.

Similarly, the percentages of responders with a 25% reduction in NIH-CPSI pain score were similar to the percentages of total NIH-CPSI score responders, both NMT group (65.05% at 4 weeks, P = 0.002, 82.52% at 8 weeks, *P* < 0.001) and combination group (69.70% at 4 weeks, *P* < 0.001, 84.85% at 8 weeks, *P* < 0.001) were significantly greater than those who received tamsulosin (43.01% at 4 weeks, 51.61% at 8 weeks).

#### 3.3.4 Subgroup analyses

**FIGURE 3:**
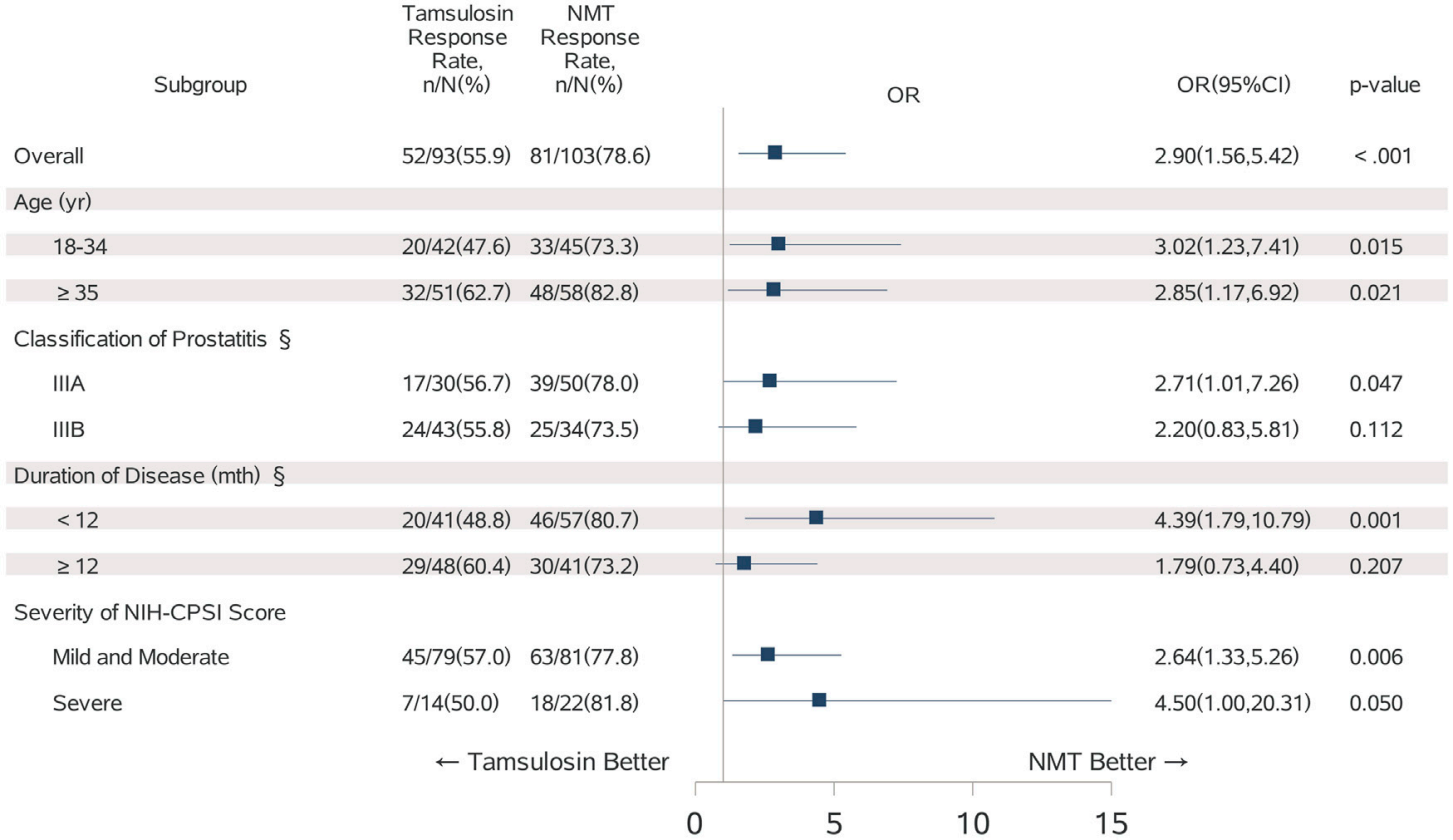
Forest Plot for Tamsulosin VS. NMT Group. §39 participants had unknown classification of prostatitis; 9 participants had unknown duration of disease; The odds ratio (shown as square in the plot) and 95%CI (shown as line interval that go through the square) were obtained in Logistic Regression; P-value was computed using Wald-Test; Odds ratio close to 0: Tamsulosin group has better response rate of 25% reduction in NIH-CPSI Total score then NMT group; Odds ratio close to 1: No difference in the response rate of 25% reduction in NIH-CPSI Total score between Tamsulosin and NMT group; Odds ratio away from 1: NMT group has better response rate of 25% reduction in NIH-CPSI Total score then Tamsulosin group.

A more in-depth analysis of therapeutic characteristics for NMT compared to tamsulosin was conducted. Overall, the odds for achieving a positive outcome were significantly higher in the NMT group than in the tamsulosin group (OR = 2.90, *P* < 0.001) (Figure 3). Subgroup models that also showed significantly higher odds for the NMT group compared to the tamsulosin group included: age (18–34 years old, OR = 3.02, P = 0.015; ≥35 years old, OR = 2.85, *P* = 0.021), classification of prostatitis (IIIA, OR = 2.71, *P* = 0.047), duration of disease (<12 months, OR = 4.39, *P* = 0.001), and severity of baseline NIH-CPSI total score (mild and moderate, OR = 2.64, *P* = 0.006).

**FIGURE 4:**
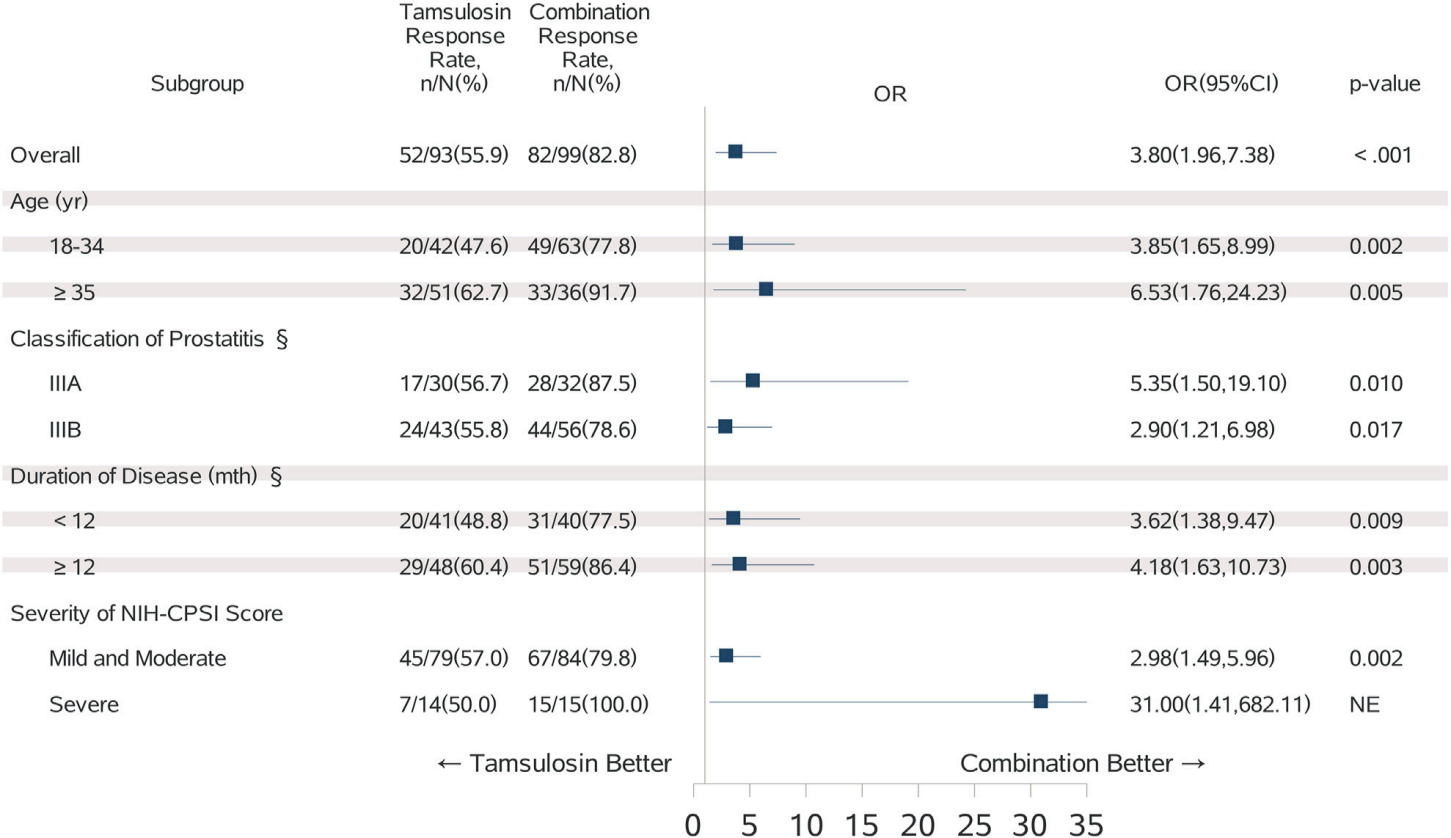
Forest Plot for Tamsulosin VS. Combination Group. §31 participants had unknown classification of prostatitis; 4 participants had unknown duration of disease; The odds ratio (shown as square in the plot) and 95%CI (shown as line interval that go through the square) were obtained in Logistic Regression; P-value was computed using Wald-Test; Odds ratio close to 0: Tamsulosin group has better response rate of 25% reduction in NIH-CPSI Total score then Combination group; Odds ratio close to 1: No difference in the response rate of 25% reduction in NIH-CPSI Total score between Tamsulosin and Combination group; Odds ratio away from 1: Combination group has better response rate of 25% reduction in NIH-CPSI Total score then Tamsulosin group; NE: Not Evaluable.

Furthermore, logistic regression was performed in the same subgroups to assess the odds ratio trend for the combination group compared to the tamsulosin group (Figure 4). The results indicated that the odds of the combination group were significantly higher than the tamsulosin group, not only in the overall population (OR = 3.80, *P* < 0.001), but also consistently across all analyzed subgroups (all OR >1 and *P* < 0.05).

### 3.4 Adverse events

In the Safety Set (SS, n = 323), a total of 7 subjects reported at least one adverse event (AE). One subject (0.93%) in the tamsulosin group reported three AEs, consisting of dizziness (twice) and nasal congestion (once). One subject (0.92%) in the NMT group reported a single AE of stomach discomfort. In the combination group, five subjects (4.72%) reported a total of eight AEs, including stomach discomfort (four times), dizziness (twice), abnormal penile erection (once), and burnout (once). There was no statistically significant difference in the incidence of adverse reactions among the three treatment groups. Importantly, no serious adverse events (SAEs) were reported throughout the entire study period, and no patients withdrew from the study due to AEs (Table 6).

**TABLE 6:** Incidence of adverse events.

Variable	Tamsulosin group (N = 108)	NMT group (N = 109)	Combination group (N = 106)
Adverse events (AEs)	3	1	8
Subjects with AEs (%)	1 (0.93%)	1 (0.93%)	5 (4.72%)
AEs reported			
Dizziness	2		2
Nasal congestion	1		
Stomach discomfort		1	4
Abnormal penile erection			1
Burnout			1

Furthermore, liver function, as assessed by AST and ALT levels, remained within the normal range for all subjects at both baseline and 8 weeks, with no significant differences observed among the three groups (Table 7).

**TABLE 7:** Levels of AST and ALT at baseline and 8 weeks.

Group	AST (U/L)					ALT (U/L)				
	Baseline		8 Weeks			Baseline		8 Weeks		
	N	Means ± SD	N	Means ± SD	P-value^a^	N	Means ± SD	N	Means ± SD	P-value^a^
Tamsulosin	27	26.80 ± 6.01	30	25.70 ± 5.77	0.6700	27	29.90 ± 13.01	30	27.19 ± 10.19	0.4886
NMT	25	26.16 ± 6.97	25	25.87 ± 4.94	0.7501	25	30.13 ± 13.92	25	26.44 ± 9.15	0.1194
Combination	53	30.19 ± 7.15	51	31.61 ± 8.11	0.3738	54	36.28 ± 13.53	51	36.94 ± 12.11	0.8080

^a^ Paired-t test was used to test mean difference before and after treatment.

ALT: alanine aminotransferase, AST: aspartate aminotransferase.

NMT: ningmitai capsule, Combination: Ningmitai capsule+tamsulosin.

## 4 Discussion

This multicenter, randomized, positive-controlled trial provides preliminary evidence regarding the efficacy and safety of NMT in patients with abacterial CP/CPPS. The major findings demonstrate that: 1. combination therapy of NMT and tamsulosin resulted in a significantly greater and clinically meaningful reduction in overall CP/CPPS symptoms, as measured by the NIH-CPSI total score, compared to tamsulosin monotherapy; 2. NMT alone demonstrated superior improvements in NIH-CPSI total score, pain, and QoL subdomains compared to tamsulosin; and 3. NMT was well-tolerated with a favorable safety profile across the 8-week treatment period.

Alpha-blockers have often been prescribed for men with CP/CPPS for the reason that they are considered first-line treatment for lower urinary tract symptoms which could reduce neurogenic inflammation in the lower urinary tract (Geppetti et al., 2008). Their mechanism involves relaxing the smooth muscles in the prostate and bladder neck, which can improve urinary flow and alleviate pain related to voiding dysfunction (Widia et al., 2023). However, clinical evidence regarding their effectiveness in CP/CPPS remains inconsistent. For instance, a large trial involving 196 subjects evaluating 6 weeks of tamsulosin therapy showed no significant benefit on the total NIH-CPSI score (Alexander et al., 2004). Similarly, another study in 272 alpha-blocker-naive CP/CPPS patients found no significant clinical or statistical benefit from 12 weeks of alfuzosin therapy compared to placebo (Nickel et al., 2008). Same as these observations, our present study revealed that after an 8-week course of tamsulosin therapy, only 55.91% of Chinese CP/CPPS patients, with an average disease duration of 18 months and regardless of prior alpha-blocker exposure, achieved symptom amelioration defined as a ≥25% reduction in NIH-CPSI score. This highlights the limitations of monotherapy with alpha-blockers for many CP/CPPS patients.

Besides alpha-blockers, Non-steroidal anti-inflammatory drugs (NSAIDs) are commonly used for pelvic pain. A Cochrane review indicated that NSAIDs might effectively reduce prostatitis symptoms compared to placebo (Franco et al., 2019). However, the potential for long-term side-effects, such as gastrointestinal complications, remains a significant concern, often leading patients to prioritize a higher pain threshold over the risk of adverse events. Additionally, while empirical antibiotic therapy is widely applied, with some evidence suggesting a reduction in prostatitis symptoms, the imperative to avoid unnecessary antibiotic use due to concerns about antimicrobial resistance underscores the need for alternative, non-antibiotic treatments. Meanwhile, various other pharmacological agents are sometimes employed for CP/CPPS. Neuromodulators, such as gabapentin and pregabalin, are occasionally used to target neuropathic pain components, though their efficacy can be inconsistent and they often carry side effects like dizziness and somnolence (Bashford et al., 2021). Antidepressants, including tricyclic antidepressants (TCAs) and selective serotonin reuptake inhibitors (SSRIs), are also prescribed for their analgesic and anxiolytic properties, which can aid in managing chronic pain and associated psychological distress. However, these agents have their own adverse effect profiles and frequently necessitate careful dose titration (Bair et al., 2003). Furthermore, various phytotherapy agents and complementary therapies, ranging from pollen extracts to acupuncture, have been explored, with some showing promise but often lacking large-scale clinical trial evidence (Pan et al., 2023; Cai et al., 2017). The diverse and often unsatisfactory outcomes associated with these varied monotherapies underscore the persistent challenges in CP/CPPS management. In our study, NMT’s multi-component, multi-target approach, supported by our findings demonstrating superior improvements in pain and Quality of Life compared to tamsulosin, presents a potential alternative or adjunctive therapy for CP/CPPS patients.

It should also be noted that the dosage of tamsulosin in this study was 0.2 mg once daily, which is standard in Asian populations but lower than the 0.4 mg dose commonly used elsewhere. This difference raises the possibility that the efficacy of tamsulosin may have been underestimated, and future studies should examine whether higher doses yield different comparative outcomes. Furthermore, the absence of a placebo arm or comparator treatments such as NSAIDs or antibiotics limits the ability to fully contextualize the efficacy of NMT within broader therapeutic strategies. Additional well-designed studies incorporating these comparators are needed to more comprehensively define NMT’s role in the management of CP/CPPS.

Another consideration is the relatively short 8-week follow-up period. CP/CPPS is a chronic condition that often requires long-term management, and thus the durability of symptom improvement, relapse rates, and long-term safety of NMT and combination therapy remain uncertain. Longer follow-up periods will be essential in future studies to confirm sustained efficacy and safety profiles.

TCM is widely utilized in China, with modern TCM research increasingly employing scientific methods to validate traditional herbal remedies. NMT, a standardized herbal extract mixture, has been used in China for the treatment of CP/CPPS for nearly 30 years. Its potential mechanisms have been explored through network pharmacological studies, which suggest that NMT’s 47 active ingredients, including quercetin, kaempferol, and beta-sitosterol, may inhibit the PI3K/Akt and MAPK signaling pathways, thereby disrupting the synthesis of proinflammatory cytokines (Luo et al., 2025). Further mechanistic studies have demonstrated that NMT can reduce CCL2 secretion by inhibiting MAPK, NF-κB, and STAT3 pathways, decrease substance *P* expression in the spinal dorsal root ganglion, and inhibit malondialdehyde (MDA) expression, collectively relieving inflammation, pain, and oxidative stress *in vivo* (Chen et al., 2020). These multi-component, multi-target, and multi-mechanism properties of NMT are likely beneficial for patients with CP/CPPS, aligning with the complex nature of the syndrome. Prior clinical research, including a prospective, randomized, double-blind, placebo-controlled trial, has also shown that NMT significantly improved NIH-CPSI pain and total scores, as well as QoL scores, over 2 and 4 weeks of treatment compared to placebo (Zhang et al., 2021). Notably, recent multicenter randomized controlled trials have further investigated NMT in combination with sildenafil for CP/CPPS patients with comorbid erectile dysfunction, demonstrating superior pain symptom amelioration and improved erectile function compared to monotherapies, with a favorable tolerability profile and without the orthostatic hypotension risk associated with combining PDE5i with alpha-blockers (Luo et al., 2025). In summary, NMT provide promising therapeutic benefits for CP/CPPS patients, particularly in alleviating pain, inflammation, and oxidative stress while improving quality of life.

This study represents the first comparative trial of NMT against an alpha-blocker in a large, well-defined CP/CPPS patient cohort. All treatment groups demonstrated significant symptomatic improvement over the 8-week period, as measured by the NIH-CPSI total score and its subdomains. Crucially, the NMT monotherapy group achieved significantly greater improvement in the NIH-CPSI total score, pain, and QoL subdomains compared to the tamsulosin group. The combined NMT and tamsulosin therapy further enhanced benefits, particularly in the micturition and QoL subdomains, highlighting the potential synergy of multimodal approaches. A 4-point decrease in the NIH-CPSI score is widely recognized as the minimal clinically significant difference (MCID) detectable by patients (Nickel et al., 2004; Propert et al., 2006). In our study, the responder rates (defined as ≥25% improvement in NIH-CPSI total score and/or pain subscore) were significantly higher in both the NMT group and the combination group compared to the tamsulosin group. Interestingly, subgroup analyses indicated that NMT’s treatment effect appeared superior to tamsulosin regardless of age, particularly in men with mild to moderate CP/CPPS, those with disease duration within 12 months, or those with Category IIIA (inflammatory) prostatitis. The superior efficacy of NMT in Category IIIA patients is particularly noteworthy and may be attributed to its documented anti-inflammatory effects. The consistent superiority of combination therapy over tamsulosin alone across all subgroups further supports the principle that multimodal treatment approaches are beneficial for CP/CPPS patients. Furthermore, NMT demonstrated a favorable safety profile and was generally well-tolerated throughout the entire study period, with no serious adverse events reported. These comparative findings strengthen the evidence for NMT’s clinical utility and its role in a personalized, multimodal treatment for CP/CPPS.

Despite the valuable insights gained from this study, several limitations should be considered. First, the 8-week observation period may not fully capture the long-term efficacy or durability of the treatment effects, particularly given the chronic nature of CP/CPPS. Longer follow-up studies are necessary to evaluate the sustained benefits and potential recurrence of symptoms. Second, the open-label design poses a significant limitation. Although investigators, outcome assessors, and statisticians were blinded, participant blinding was not feasible due to the distinct formulations and appearance of NMT capsules and tamsulosin tablets. This lack of participant blinding could have introduced placebo and expectation biases, particularly affecting subjective, patient-reported outcomes such as pain perception and QoL. While the consistency of improvements across multiple efficacy domains lends support to the robustness of our findings, we acknowledge that these biases cannot be fully excluded. Implementing a fully double-blind, double-dummy, placebo-controlled trial for distinct drug formulations such as NMT and tamsulosin posed considerable logistical and resource challenges in the current multicenter setting. Nevertheless, future research should prioritize efforts to adopt more rigorous blinding methodologies whenever feasible to further enhance the objectivity of findings and minimize expectancy effects. Third, the standard dosage of tamsulosin used in this study (0.2 mg/day) may not represent its maximal therapeutic potential, as higher doses are commonly used in other regions. Future studies should explore whether different doses of tamsulosin yield different outcomes, particularly when compared to NMT. Finally, this study focused on a Chinese male population, and as such, the generalizability of the results to other ethnic groups and regions may be limited. TCM therapies, including the formulation used in this study, are widely used in China and some other East Asian countries but are less commonly adopted in Western populations. Cultural attitudes toward TCM and its perceived efficacy may vary, which could influence patient adherence and treatment outcomes. Future studies should explore the applicability of NMT and similar TCM therapies in diverse populations to better understand their potential in global healthcare settings. Additionally, research should also consider the interaction of these therapies with other culturally preferred treatments in non-Chinese populations.

## 5 Conclusion

In conclusion, this multicenter, randomized, positive-controlled trial demonstrates that 8 weeks of NMT treatment effectively ameliorates symptoms in men with mild to severe abacterial CP/CPPS. This clinical benefit was primarily driven by a significant reduction in pain symptomatology and improvement in quality of life. Furthermore, the combination of NMT and tamsulosin was shown to further enhance these clinical benefits, particularly in overall symptom reduction and micturition function. Importantly, NMT was well tolerated, with no serious adverse events observed during the trial, supporting its favorable safety profile. The findings of this study provide robust initial support for NMT as a valuable therapeutic option for abacterial CP/CPPS, either as a standalone treatment or as part of a multimodal regimen, contributing to improved symptom management and patient quality of life.

## Data Availability

The original contributions presented in the study are included in the article/Supplementary Material, further inquiries can be directed to the corresponding author.

## Supplementary material

SUPPLEMENTARY FIGURE S1 Total ion current (TIC) chromatograms of NMT.

## Abbreviations

ALT, alanine aminotransferase; ANCOVA, Analysis of Covariance; AST, aspartate aminotransferase; CP/CPPS, Chronic prostatitis/chronic pelvic pain syndrome; FAS, full analysis set; GCP, Good Clinical Practice; IRT, Interactive Response Technology; ICH, the International Council for Harmonisation of Technical Requirements for Pharmaceuticals for Human Use; MAPK, mitogen-activated protein kinase; MCID, the minimal clinically significant difference; MDA, malondialdehyde; MedDRA, The Medical Dictionary for Regulatory Activities; NF-κB, nuclear transcription factor-κB; NIH-CPSI, National Institutes of Health Chronic Prostatitis Symptom Index; NMT, Ningmitai capsule; NSAIDs, Non-steroidal anti-inflammatory drugs; PPS, per protocol set; SS, safety analysis set; STAT3, Signal transducer and activator of transcription 3; TCM, traditional Chinese medicine.
