# Supplementary figures and images for: Ningmitai capsule in patients with chronic prostatitis/chronic pelvic pain syndrome: a multicenter, prospective, randomized, parallel, positive-controlled study
Source: Front Pharmacol. 2025 Dec 3;16:1667819. doi: 10.3389/fphar.2025.1667819 (PMC12708510; doi:10.3389/fphar.2025.1667819)

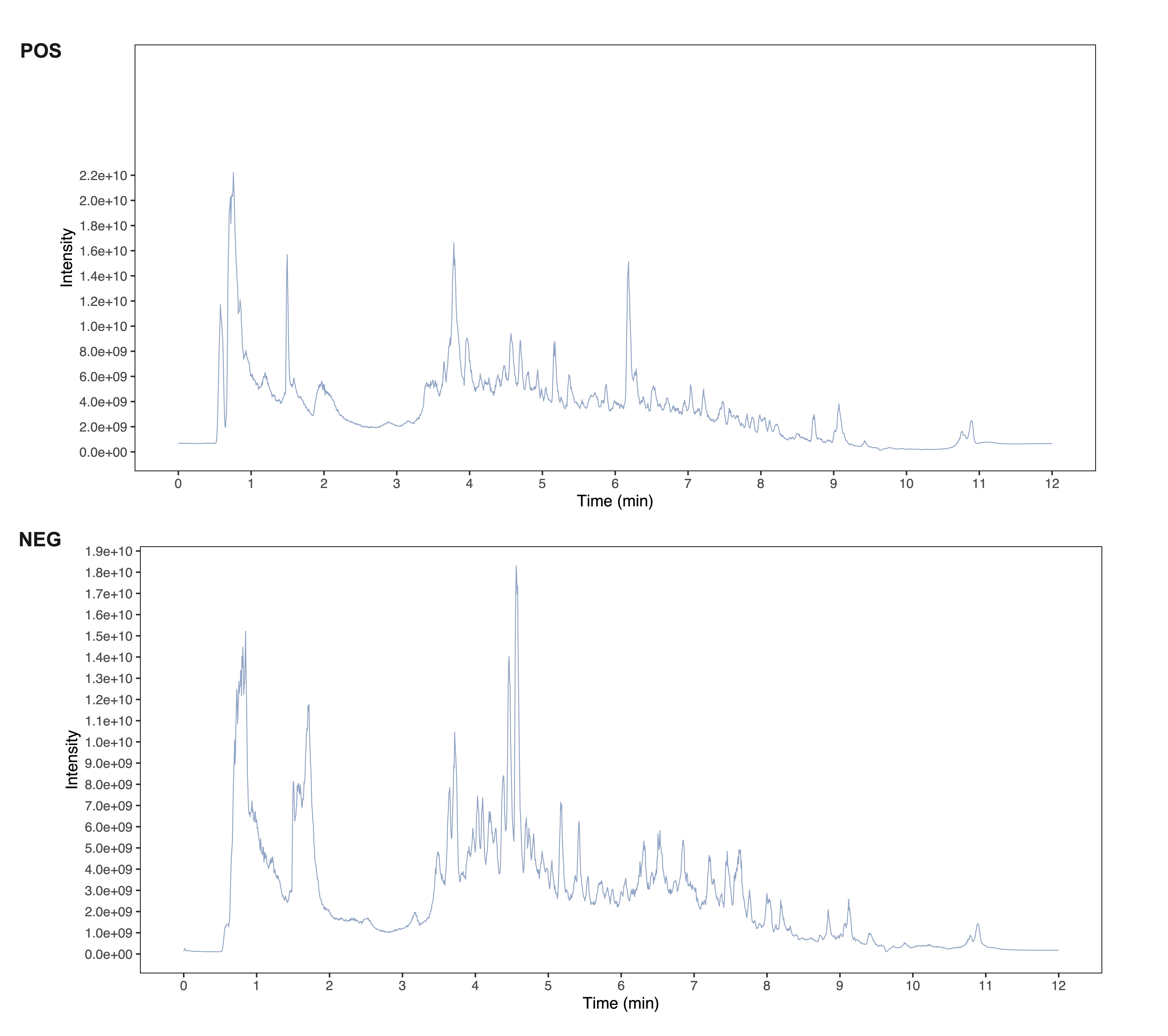

Supplement: Supplementary file 1 [file Image1.jpeg]
